# Risk of adverse events following the initiation of antihypertensives in older people with complex health needs: a self-controlled case series in the United Kingdom

**DOI:** 10.1093/ageing/afad177

**Published:** 2023-09-16

**Authors:** Annika M Jödicke, Eng Hooi Tan, Danielle E Robinson, Antonella Delmestri, Daniel Prieto-Alhambra

**Affiliations:** Pharmaco- and Device Epidemiology, Centre for Statistics in Medicine, Nuffield Department of Orthopaedics, Rheumatology and Musculoskeletal Sciences, University of Oxford, OX37LD, Oxford, UK; Pharmaco- and Device Epidemiology, Centre for Statistics in Medicine, Nuffield Department of Orthopaedics, Rheumatology and Musculoskeletal Sciences, University of Oxford, OX37LD, Oxford, UK; Pharmaco- and Device Epidemiology, Centre for Statistics in Medicine, Nuffield Department of Orthopaedics, Rheumatology and Musculoskeletal Sciences, University of Oxford, OX37LD, Oxford, UK; Pharmaco- and Device Epidemiology, Centre for Statistics in Medicine, Nuffield Department of Orthopaedics, Rheumatology and Musculoskeletal Sciences, University of Oxford, OX37LD, Oxford, UK; Pharmaco- and Device Epidemiology, Centre for Statistics in Medicine, Nuffield Department of Orthopaedics, Rheumatology and Musculoskeletal Sciences, University of Oxford, OX37LD, Oxford, UK; Department of Medical Informatics, Erasmus Medical Center University, 40 3015 GD, Rotterdam, Netherlands

**Keywords:** self-controlled case series, antihypertensives, drug safety, pharmacoepidemiology, acute kidney injury, falls, older people

## Abstract

**Background:**

We assessed the risk of adverse events—severe acute kidney injury (AKI), falls and fractures—associated with use of antihypertensives in older patients with complex health needs (CHN).

**Setting:**

UK primary care linked to inpatient and mortality records.

**Methods:**

The source population comprised patients aged >65, with ≥1 year of registration and unexposed to antihypertensives in the year before study start. We identified three cohorts of patients with CHN, namely, unplanned hospitalisations, frailty (electronic frailty index deficit count ≥3) and polypharmacy (prescription of ≥10 medicines). Patients in any of these cohorts were included in the CHN cohort. We conducted self-controlled case series for each cohort and outcome (AKI, falls, fractures). Incidence rate ratios (IRRs) were estimated by dividing event rates (i) during overall antihypertensive exposed patient-time over unexposed patient-time; and (ii) in the first 30 days after treatment initiation over unexposed patient-time.

**Results:**

Among 42,483 patients in the CHN cohort, 7,240, 5,164 and 450 individuals had falls, fractures or AKI, respectively. We observed an increased risk for AKI associated with exposure to antihypertensives across all cohorts (CHN: IRR 2.36 [95% CI: 1.68–3.31]). In the 30 days post-antihypertensive treatment initiation, a 35–50% increased risk for falls was found across all cohorts and increased fracture risk in the frailty cohort (IRR 1.38 [1.03–1.84]). No increased risk for falls/fractures was associated with continuation of antihypertensive treatment or overall use.

**Conclusion:**

Treatment with antihypertensives in older patients was associated with increased risk of AKI and transiently elevated risk of falls in the 30 days after starting antihypertensive therapy.

## Key Points

- Treatment with antihypertensives in older patients with complex health needs was associated with increased risk of acute kidney injury.
- Risk of falls was transiently elevated in the 30 days after starting antihypertensive therapy.
- No increased risk for falls and fractures was associated with overall antihypertensive treatment exposure in this patient group.

## Introduction

Global population ageing is projected to grow rapidly from 461 million people aged at least 65 years to 2 billion by 2050 [1]. Age is strongly associated with multimorbidity [1] and emergency hospital admission [2]. Among this older population, those with frailty, frequent hospital admission and polypharmacy are at greater risk of long-term care, disability and mortality [1].

Hypertension is highly prevalent in older people, particularly among the frail. A recent population-based study in the United Kingdom reported increased prevalence of hypertension from 66 to 76% with higher severity of frailty [3]. Given the vulnerability of older people to adverse events, it is a challenge to balance the benefit of initiating antihypertensives and the risk of encountering serious side effects such as fall-related injuries [4, 5] and acute kidney injury (AKI) [6]. Falls and fractures increase chronic pain, disability and mortality [7], whereas AKI leads to chronic kidney disease [8] and poorer cardiovascular outcomes [9]. Studies have shown that overlapping factors such as polypharmacy, frailty and specific drugs, including antihypertensives, predispose older people to falls, fractures and acute renal failure [10]. However, estimates of adverse effects can be influenced by residual confounding. For instance, patients indicated for antihypertensives can also have multiple comorbidities which elevate risk of adverse events. The design of self-controlled case studies (SCCS) [11] has been used to overcome this limitation by adjusting for time-invariant confounding as individuals are analysed as their own control.

Therefore, our study objective was to assess the effects of antihypertensives on an older population with complex health needs, namely, unplanned admissions, frailty and polypharmacy.

## Methods

### Data source

The Clinical Practice Research Datalink (CPRD GOLD) is a primary care dataset comprising anonymised electronic health records for >17 million patients in the United Kingdom [12]. The dataset is representative of the UK population and includes information on patient demographics, conditions, drug prescriptions, laboratory tests, health-related lifestyle factors and secondary care referrals [13]. For this study, we linked CPRD GOLD [14] to Hospital Episode Statistics (HES) inpatient data, the Office for National Statistics mortality data and the Index of Multiple Deprivation for patient-level socioeconomic status.

### Study population

The source population comprised all patients aged >65 years at study start (1 January 2010), who were registered with an ‘up-to-standard’ practise [13] for ≥1 year and could be linked to HES. We excluded patients who were exposed to antihypertensives in the year before study start. A minimum follow-up duration of 1 day was required.

**Figure 1:**
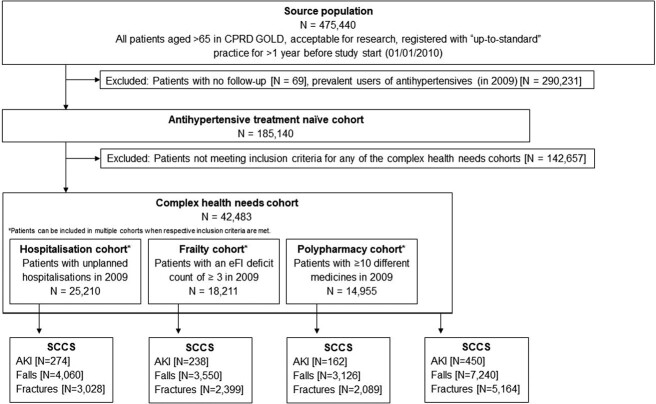
Study inclusion flowchart. *N* = number of individuals, CPRD = Clinical Practice Research Datalink, eFI = electronic Frailty Index. Unplanned hospitalisations were defined as unplanned admission via Admitted Patient Care or admissions through the Accident and Emergency department in HES.

Subsequently, we identified patients with complex health needs (CHN) based on three different indicators assessed in the year before study start [15]: patients with unplanned hospitalisations (i.e. admissions that are unpredictable and emergency admissions from HES) were included to the hospitalisation cohort; patients with an electronic frailty index deficit count [16] of ≥3 constituted the frailty cohort; and patients with ≥10 different medicines prescribed comprised the polypharmacy cohort. All patients included in any of these three cohorts were automatically included to the CHN cohort to increase sample size. A study inclusion flowchart is provided in Figure 1.

### Outcomes

Outcomes of interest were falls, fractures and severe AKI. Falls were identified from CPRD based on recorded READ codes (a coded thesaurus of clinical terms), whereas fractures were identified from both CPRD (READ codes) and from main hospital diagnosis in HES (ICD-10 codes) (Appendix 2). AKI was identified based on ICD-10 N17 (‘acute renal failure’) and N19 (‘unspecified kidney failure’) codes [17, 18] in HES. Multiple recordings of the respective outcomes within 30 days were considered duplicates and removed from the dataset. Fractures were considered both overall and stratified for type, namely, hip, vertebral and non-hip–non-vertebral fractures. For sensitivity analysis, only fractures identified from hospital records were considered.

### Exposure

Antihypertensives comprised the following drug classes as categorised according to the World Health Organisation [19]: angiotensin-converting-enzyme inhibitors (ACE-I), angiotensin-II receptor blockers (ARB), beta blockers (BB), calcium channel blockers (CCB), diuretics, renin inhibitors and other antihypertensives, as well as their respective combinations. For this study, we analysed antihypertensives as a group, and separately for ACE-I, ARB, BB, CCB and diuretics.

Prescriptions were identified based on product-specific codes in CPRD (Appendix 2). For each patient, exposure started on the date of the first prescription for all antihypertensives and for the different antihypertensive classes, respectively. Durations for individual prescriptions were estimated from CPRD, with exposure periods being constructed based on the method proposed by Gardarsdottir *et al*. [20]: Exposure was based on periods of continuous drug use, defined as a maximum refill gap of 90 days between repeat prescriptions. A gap length of 180 days was used for sensitivity analysis. To account for non-compliance and stockpiling, a grace period of 90 days was added to the end of each exposure period.

### Study design

We conducted SCCS analyses to assess risk for adverse events (AEs)—namely, AKI, falls and fractures—associated with antihypertensives. The SCCS is an intra-individual study design, which compares event rates between exposed and unexposed patient time among patients who experienced the event of interest during follow-up [11, 21, 22]. End of follow-up was defined as the earliest of patient leaving practise, practise last data collection date, end of HES Admitted Patient Care linkage coverage, death [23] or date of data extraction (13 September 2019). We assessed risk for AE associated with (i) overall antihypertensive exposure and (ii) treatment initiation, with splitting the exposed time following antihypertensive treatment start into risk windows (0–30 days and 31+ days for falls/fractures; 0–30 days, 31–90 days, 91+ days for AKI). The study design is illustrated in Appendix 1Figure S1.

### Statistical analyses

Descriptive statistics were provided separately for each cohort. Frequencies were provided for categorical variables, whereas median and interquartile range were provided for continuous variables.

SCCS were conducted separately for each outcome and for each cohort of patients with CHN. Incidence rate ratios (IRRs) were derived using conditional Poisson regression and calculated in R (version 4.0.3, SCCS package [24]). First, we compared event rates for overall exposed and unexposed period. Second, we separately compared event rates for each risk window to the unexposed period. Age was adjusted for in 1-year age bands for falls and fractures, and in 2-year age bands for AKI (due to smaller event frequencies).

We conducted sensitivity analyses to test the robustness of our results with respect to the assumptions of the SCCS methods [11]: we (i) excluded patients who died during follow-up, (ii) used only the first recording of each respective event per patient, (iii) included a 30-day pre-exposure period before start of antihypertensive and (iv) excluded patients with history of the respective outcomes. Where sample size allowed, we stratified our analyses for antihypertensive drug classes.

## Results

Among 185,140 antihypertensive-naïve patients, 42,483 were included to the CHN cohort hospitalisation (25,210), frailty (18,211) and polypharmacy (14,955). Of these, 7,240 (17.0%) had at least one fall recorded during follow-up, whereas 5,164 individuals (12.2%) had at least one fracture, and 450 (1.1%) individuals experienced an incident of AKI. Demographic characteristics for the CHN cohort are presented in Table 1. Additional characteristics for each cohort are described in Appendix 1Table S1.

**Table 1:** Patient characteristics of complex health needs cohort, stratified for outcome

	**Fall**	**Fracture**	**AKI**
*N*	7,240	5,164	450
Age, years	79 [73, 84]	78 [72, 84]	79 [73, 85]
Gender: female	4,860 (67%)	3,874 (75%)	209 (46%)
Follow-up, years	5.1 [3.3, 6.6]	5.3 [3.3, 6.7]	4.3 [2.2, 6.3]
Antihypertensive exposure during follow-up	3,347 (46%)	2,303 (45%)	263 (58%)
Treatment duration, years	1.7 [0.6, 3.6]	1.7 [0.5, 3.5]	1.7 [0.6, 3.6]
Survival: died	1,905 (26%)	1,408 (27%)	273 (61%)
Number of events			
1	5,173 (71%)	4,092 (79%)	417 (93%)
2	1,337 (18%)	831 (16%)	26 (5.8%)
≥3	730 (10%)	241 (4.7%)	7 (1.6%)
History of outcome event,* anytime	2,675 (37%)	1,580 (31%)	22 (4.9%)
Socioeconomic status			
1 (Most deprived)	1,749 (24%)	1,262 (24%)	98 (22%)
2	1,818 (25%)	1,260 (24%)	118 (26%)
3	1,527 (21%)	1,102 (21%)	110 (24%)
4	1,275 (18%)	924 (18%)	65 (14%)
5 (least deprived)	864 (12%)	613 (12%)	59 (13%)
Body mass index			
Underweight	299 (4.1%)	306 (5.9%)	15 (3.3%)
Normal	2,399 (33%)	1,741 (34%)	126 (28%)
Overweight	1,850 (26%)	1,227 (24%)	121 (27%)
Obese	867 (12%)	541 (10%)	52 (12%)
Drinking status, 5 yr			
Drinker	413 (5.7%)	375 (7.3%)	36 (15%)
Ex-drinker	1,423 (20%)	918 (18%)	96 (41%)
Non-drinker	2,012 (28%)	1,365 (26%)	105 (44%)
Smoking status, 5 yr			
Smoker	737 (10%)	661 (13%)	60 (15%)
Ex-smoker	2,415 (33%)	1,616 (31%)	161 (40%)
Non-smoker	3,507 (48%)	2,460 (48%)	184 (45%)
Systolic BP, 1 yr, mmHg	135 [124, 143]	135 [124, 144]	136 [126, 144]
Diastolic BP, 1 yr, mmHg	76 [70, 80]	78 [70, 81]	76 [70, 80]
No. of medications, 1 yr			
<10	4,114 (57%)	3,075 (60%)	298 (66%)
10–15	2,326 (32%)	1,616 (31%)	113 (25%)
>15	800 (11%)	473 (9.2%)	49 (11%)
Electronic frailty index (eFI) deficit count, 1 yr	2 [1, 3]	2 [1, 3]	3 [1, 3]
General practitioner visits, 1 yr	11 [7, 18]	11 [6, 17]	11 [7, 18]
History of comorbidities, anytime			
Diabetes	715 (9.9%)	451 (8.7%)	76 (17%)
Vascular disease**	1,087 (15%)	655 (13%)	72 (16%)
Chronic renal failure	943 (13%)	607 (12%)	110 (24%)
Osteoporosis	1,086 (15%)	969 (19%)	45 (10%)

Statistics are presented in *N* (%) and median [interquartile range]. Missingness of covariates for patients: index of multiple deprivation, <0.1%; BMI, 19–37%; drinking status, 41–50%; smoking status, 3–13%; systolic BP, 17–39%; diastolic BP, 17–38%. BP = blood pressure, ACE-I = angiotensin-converting enzyme inhibitor, ARB = angiotensin-II blocker, BB = beta blocker, CCB = calcium channel blocker.

^a^Falls, fractures and AKI, respectively.

^b^Vascular disease include angina pectoris, artery occlusion, atherosclerosis, heart failure, ischaemic heart disease, myocardial infarction, peripheral vascular disease, stroke and transient ischaemic attack.

### Cohort characteristics

#### Falls

Patients with falls in the CHN cohort had median age of 79 years and were predominantly females (67%). Among those who started antihypertensive treatment, diuretics were prescribed most frequently, with prescriptions being recorded in 63% of patients anytime during follow-up. CCBs were prescribed in 35% of patients, followed by ACE-I (32%), BB (30%) and ARB (9%).

#### Fractures

Patients with fractures had median age of 78 years and were predominantly females (75%). Among individuals with fractures, non-hip–non-vertebral fractures were the most common fracture type, with 78% of patients experiencing at least one during follow-up. Hip and vertebral fractures were less common, with 32% and 9% of patients having at least one respective recording during follow-up. Diuretics were prescribed most frequently, with prescriptions being recorded in 63% of patients anytime during follow-up. CCBs were prescribed in 35% of patients, followed by ACE-I (31%), BB (30%) and ARB (8%).

#### Acute kidney injury

Patients with AKI had median age of 79 years and were less predominantly females (46%). There were 24% of patients with history of chronic renal failure. Diuretics were prescribed most frequently, with prescriptions being recorded in 60% of patients anytime during follow-up. ACE-I were prescribed in 43% of patients, followed by CCBs (37%), BB (37%) and ARB (8%).

### SCCS for falls

**Figure 2:**
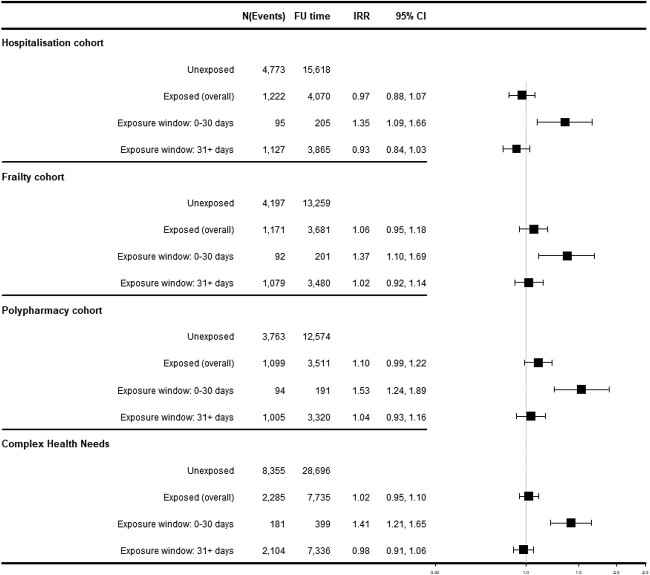
SCCS for falls. *N* = number of events, FU = follow-up in patient years, 95% CI = 95% confidence interval.

A 35–50% increased risk for falls associated with antihypertensive treatment initiation was observed across all cohorts, while no increased risk was found for either the overall exposed period or the 31 days onwards (Figure 2). Sensitivity analyses using (i) only the first fall per patient and (ii) excluding patients who died during follow-up showed increased fall risk in the polypharmacy cohort. Results from all other sensitivity analyses were consistent with the main findings for the 30-day period for all cohorts (Appendix 1Table S2). Stratification for antihypertensive drug classes resulted in very small event counts, which did not allow for robust IRR estimations (Appendix 1Table S3).

### SCCS for fractures

**Figure 3:**
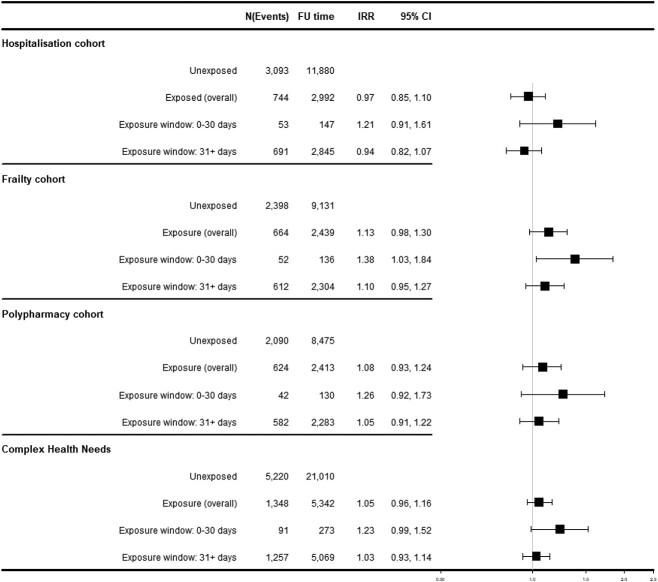
SCCS for fractures. *N* = number of events, FU = follow-up in patient years, 95% CI = 95% confidence interval.

Increased risk for any fracture in the 30 days following antihypertensive treatment initiation was observed for the frailty cohort with IRR 1.38 [95% CI: 1.03–1.84], but no significant association was seen for the other cohorts (Figure 3). No increased fracture risk was associated with continuation of antihypertensive exposure from 31 days onwards (Figure 3). Sensitivity analyses for (i) first fracture and (ii) pre-risk window showed increased fracture risk after treatment initiation for the frailty cohort, whereas excluding patients who died during follow-up was not significant (IRR 1.41 [0.98–2.03]). Stratification for fracture type showed 13, <5 and 39 events of hip, vertebral and non-hip–non-vertebral fractures in the first 30 days after treatment start, highlighting that the main results were predominantly driven by non-hip–non-vertebral fractures (frailty cohort: IRR 1.46 [1.04–2.04]). Results from all other sensitivity analyses are provided in Appendix 1Table S4. Fracture event counts were small in antihypertensive-class stratified analyses (Appendix 1Table S5).

### SCCS for AKI

**Figure 4:**
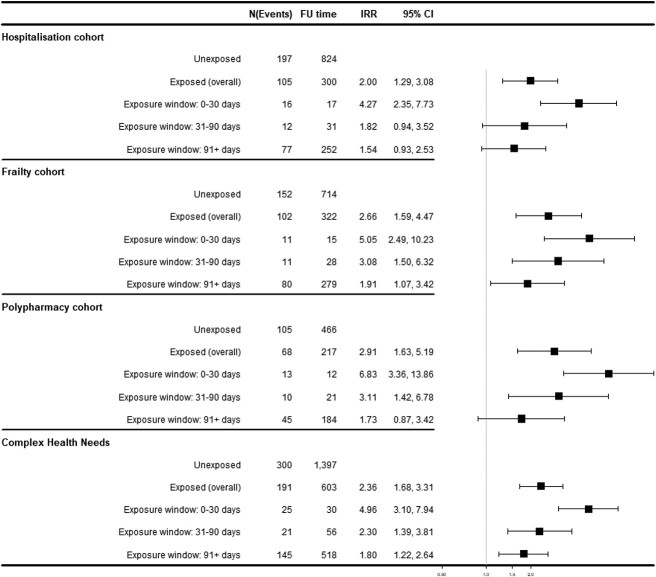
SCCS for AKI. *N* = number of events, FU = follow-up in patient years, 95% CI = 95% confidence interval.

The main SCCS model showed an increased risk of AKI for the hospitalisation cohort (IRR 2.00 [95% CI: 1.29–3.08]), frailty cohort (IRR 2.66 [1.59–4.47]), polypharmacy cohort (IRR 2.91 [1.63–5.19]) and CHN cohort (IRR 2.36 [1.66–3.81]) (Figure 4). For all cohorts, higher risk was observed in the immediate 30 days after initiation of AH. This risk was attenuated as exposure window increased to 31–90 days and beyond 90 days (Figure 4), but the number of events in the 30-day and 90-day windows was small. The sensitivity analyses were consistent with our main results (Appendix 1Table S6). Overall risks were attenuated when only patients who survived all follow-up were included: IRR 1.02 [0.54–1.91] (hospital), 1.84 [0.79–4.30] (frail) and 2.02 [0.86–4.73] (polypharmacy), although the confidence intervals were wider because of smaller sample size.

## Discussion

### Main findings

Our study suggests a 2- to 3-fold increased risk for AKI associated with exposure to antihypertensives in older people with CHN. No increased risk for falls and fractures was associated with overall exposure. However, in the immediate 30 days after initiating antihypertensives, risk for AKI was increased 4- to 7-fold and falls increased by 35–50%.

### Falls and fractures

Dizziness, balance impairment and postural hypotension are common adverse effects of antihypertensives, presenting major risk factors for falls and fractures in older people—particularly in frail individuals.

Inconsistent findings for antihypertensive-related falls and fractures in the literature highlight the complexity of this association, considering different antihypertensive drug classes, chronic use [25] or recent treatment initiation [4, 5, 26], treatment intensity [27, 28], outcome definition (e.g. falls, injurious falls) and study populations. A recent meta-analysis [6] found no increased fall risk associated with antihypertensive treatment in randomised controlled trials (RR 1.05 [95% CI 0.89–1.24]). However, clinical trials often exclude the frailest among the older population, who are at particular risk for falls and fractures. While chronic use of antihypertensives was not associated with falls in older people (OR 0.97 [0.93–1.01]) [25], several studies reported increased risk for falls [26] and injurious falls [4, 5] when initiating or re-starting antihypertensives in long-term users after short treatment breaks [29]: Gribbin and colleagues [26] assessed the risk for a first fall in the 21 days following antihypertensive treatment initiation using SCCS in older UK patients. Increased fall risk was reported for thiazide diuretics (IRR 1.63 [1.20–2.20]), but not for other antihypertensive drug classes. Another SCCS [4] among community-dwelling Canadians aged ≥66 found increased risk for injurious falls in the first 14 days and 45 days following antihypertensive treatment initiation (IRR 1.94 [1.75–2.16] and IRR 1.69 [1.57–1.81], respectively). Likewise, a case-crossover study [5] in Medicare beneficiaries aged ≥65 years observed increased odds for serious fall injury during the 15 days following antihypertensive initiation, when adding a new antihypertensive drug class and following dose titration (OR 1.36 [1.19–1.55], 1.16 [1.10–1.23] and 1.13 [1.08–1.18], respectively). These associations were attenuated beyond 15 days.

Intra-individual study designs, such as case-crossover studies or SCCS, by design account for confounders that are largely stable over time, including those that are difficult to measure in observational data, such as frailty levels. Results from our sensitivity analyses testing the SCCS method’s assumptions were consistent with the main findings, as was a subgroup analysis restricting to patients without a history of previous falls. While increased risk for falls in the immediate time after treatment initiation was observed consistently across all four cohorts, a significant increased fracture risk was only observed in the frailty cohort, with all other cohorts showing a non-significant trend towards increased fracture risk. Comparing the baseline characteristics across cohorts, prevalence of previous falls and fractures was comparable. However, previous studies have identified frailty as a significant predictor of future falls among older people living in the community [30]: Individuals with 2 or ≥3 out of 6 frailty indicators had a 1.5- to 2-fold increased risk for fall-related fractures, brain injuries or joint dislocations [31]. Likewise, frailty was associated with recurrent falls or fractures (OR 1.74 [1.19–2.55] and OR 3.09 [1.04–9.21], respectively) [32] in participants aged ≥75, whereas no association was found for ≥1 fracture (OR 1.11 [0.71–1.72]) [32].

### Acute kidney injury

The incidence of AKI is increasing, contributed by an ageing population and occurrence of multiple comorbidities such as diabetes and chronic kidney disease [33]. A meta-analysis [6] of randomised controlled trials indicated that antihypertensive treatment was associated with an 18% increased risk of AKI. Certain antihypertensives were associated with elevated risk of AKI. In two cohort studies using CPRD, Scott *et al*. [34] reported 23–24% increased risk of AKI in patients exposed to ACE-I, ARB or diuretics in comparison, whereas Mansfield *et al*. [17] found a smaller increase of 12% in patients exposed to ACE-I/ARB. The magnitude of risk found in our study was more similar to that associated with combination therapy of ACE-I and ARB reported by Mansfield *et al*. (RR 1.83 [1.53–2.17]). The small number of AKI events limited our ability to perform further investigation looking at specific drug classes and combination therapy. Nonetheless, our study utilised the SCCS method to account for time-invariant confounding, which is different from the traditional cohort design. Our population was also different as we included older patients with more CHN, which are often excluded from trials. In a trial where participants were aged 50 years and above without diabetes, intensive systolic blood pressure lowering was associated with higher rates of AKI, although the injuries were mostly mild and reversible.

### Strengths and limitations

Our study comes with strengths and limitations. We considered the use of CPRD a particular strength. CPRD has been used previously in several studies to study fracture risk and AKI [35], providing high-quality data for research. In addition, we applied the SCCS method, which allowed for accounting for in-person time-invariant confounding. To account for time-varying confounding, we adjusted our models for age. However, other confounders including changes in conditions or co-medication over time might not have been captured completely. Likewise, changes in antihypertensive doses as well as switching between antihypertensive drug classes has not been considered. Ascertainment of exposed and unexposed patient time is crucial for the SCCS design as it focuses on the timing of the respective events. As for all prescription data, no information on the actual drug dispensation and intake was available. As described in the literature, falls might be under-recorded in primary care data, as falls not leading to injury may not be recorded or recorded in free text, or the consequence of the fall (e.g. fracture) might be recorded instead. The extent of under-recording of AKI in CPRD is unknown [18]. In the absence of a relevant biomarker [36], we have included two commonly used diagnostic codes to define our outcome. Since the SCCS only included patients with events of interest, under-recording might only affect the recording of potential recurrent events. We used a 30-day washout window to exclude duplicate recordings of the same event. However, for fractures, the fracture site was not considered for overall fracture and non-hip–non-vertebral fractures, potentially leading to exclusion of new fractures occurring within 30 days. Nevertheless, sensitivity analyses considering only the first fracture, and using a longer washout period between fractures, were consistent with the finding of increased fracture risk following antihypertensive treatment initiation.

### Clinical importance

Older people, particularly those suffering from polypharmacy, frailty and multimorbidity, are especially susceptible to AE due to reduced physiological reserves and altered drug responsiveness. Rapid frailty assessment was suggested when starting antihypertensives in patients aged ≥80 years to detect when a change in treatment strategy or dose adaptations may be needed [37]. Reduced renal function is common in older, frail people and orthostatic hypotension caused by antihypertensive therapy can make them prone to syncope, fall-related injuries and fractures [37]. Consequently, renal function should be monitored when starting antihypertensives in frail people as well as careful choosing of antihypertensive drug class and initial dosing. The NICE guideline recommends monitoring renal function 1 to 2 weeks after starting ACE-I [38] or ARB [39]. The Kidney Disease Outcomes Quality Initiative recommends close monitoring of renal function in older people when more intensive systolic blood pressure below 130 mmHg is targeted [40]. Likewise, patients should be informed about possible AE associated with antihypertensive treatment initiation. Several guidelines [41, 42] for treatment of hypertension and cardiovascular diseases provide recommendations for treatment initiation, including specific risk–benefit assessments for the oldest old. Considering the high risk of cardiovascular events, comorbidities and co-medication when starting new therapies in patients with CHN is required to ensure a safe and efficient therapy as much as possible. Our findings support the recommendations of clinical guidelines to monitor risk of AKI and falls especially during initiation of therapy. We observed a reduction in risk of falls after 30 days post starting antihypertensives, suggesting this AE may be less of a concern during maintenance therapy.

## Conclusion

In this SCCS of CPRD UK data, treatment with antihypertensives in older patients was associated with increased risk of AKI and transiently elevated risk of falls in the 30 days after starting antihypertensive therapy. No increased risk for fractures was associated with overall antihypertensive treatment exposure.

## Supplementary Material

aa-22-0728-File002_afad177

## Data Availability

Analytic code available on request to the author.

## Patient and Public Involvement

The grant protocol has been reviewed by a frail patient and by two charity representatives, who all gave helpful feedback.

## Data Sharing

Data were obtained from CPRD under the Oxford University CPRD licence. Direct data sharing is not allowed. Data access can be obtained from CPRD, conditional on ISAC approval.
