# Supplementary material for: Risk of adverse events following the initiation of antihypertensives in older people with complex health needs: a self-controlled case series in the United Kingdom
Source: Age Ageing. 2023 Sep 16;52(9):afad177. doi: 10.1093/ageing/afad177 (PMC10508980; doi:10.1093/ageing/afad177)
Supplement: aa-22-0728-File002_afad177 [file aa-22-0728-file002_afad177.docx]

**Appendix 1**

Risk of adverse events following the initiation of antihypertensives in older people with complex health needs: Self-controlled case series in the United Kingdom

**Figure S1: SCCS study design schema**

**
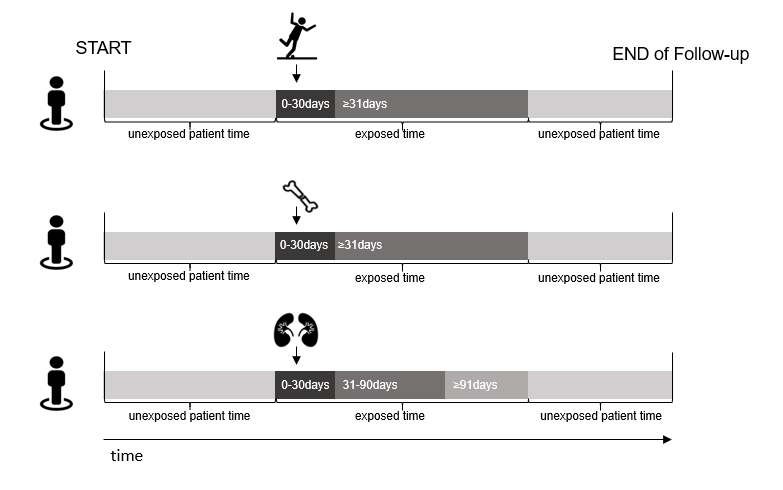
**

Self-controlled case series: (1) Risk for adverse events associated with overall AH use was assessed comparing “exposed time” and “unexposed patient time”. (2) Exposed time was subsequently split into risk windows to assess the impact of treatment initiation.

**Table S1:** Patient characteristics, stratified for outcome and cohort of complex health needs.

|  | **FALL** | | | | **FRACTURE** | | | | **AKI** | | | |
| --- | --- | --- | --- | --- | --- | --- | --- | --- | --- | --- | --- | --- |
|  | **Hospitali-sation** | **Frailty** | **Poly-pharmacy** | **Complex Health Needs** | **Hospitali-sation** | **Frailty** | **Poly-pharmacy** | **Complex Health Needs** | **Hospitali-sation** | **Frailty** | **Poly-pharmacy** | **Complex Health Needs** |
| **N** | 4,060 | 3,550 | 3,126 | 7,240 | 3,028 | 2,399 | 2,089 | 5,164 | 274 | 238 | 162 | 450 |
| **Age, years** | 79 [73, 85] | 80 [74, 85] | 78 [73, 84] | 79 [73, 84] | 78 [72, 84] | 79 [73, 85] | 78 [72, 83] | 78 [72, 84] | 80 [73, 85] | 80 [74, 85] | 79 [73, 85] | 79 [73, 85] |
| **Gender** Female | 2,627 (65%) | 2,398 (68%) | 2,221 (71%) | 4,860 (67%) | 2,194 (72%) | 1,827 (76%) | 1,650 (79%) | 3,874 (75%) | 118 (43%) | 117 (49%) | 88 (54%) | 209 (46%) |
| **Follow-up**, years | 4.8 [3.1, 6.5] | 4.7 [3.0, 6.4] | 5.3 [3.3, 6.8] | 5.1 [3.3, 6.6] | 4.9 [3.1, 6.6] | 4.9 [2.9, 6.5] | 5.3 [3.4, 7.0] | 5.3 [3.3, 6.7] | 3.8 [1.8, 5.9] | 4.2 [2.2, 6.0] | 4.0 [1.8, 6.3] | 4.3 [2.2, 6.3] |
| **Antihypertensives exposure during follow-up** | 1,756 (43%) | 1,644 (46%) | 1,537 (49%) | 3,347 (46%) | 1,283 (42%) | 1,096 (46%) | 1,044 (50%) | 2,303 (45%) | 156 (57%) | 136 (57%) | 100 (62%) | 263 (58%) |
| **Treatment**  **duration**, years | 1.7 [0.6, 3.5] | 1.6 [0.5, 3.4] | 1.6 [0.5, 3.4] | 1.7 [0.6, 3.6] | 1.6 [0.6, 3.5] | 1.6 [0.5, 3.3] | 1.6 [0.6, 3.5] | 1.7 [0.5, 3.5] | 1.4 [0.5, 2.8] | 1.9 [0.6, 3.6] | 1.5 [0.4, 3.1] | 1.7 [0.6, 3.6] |
| **Survival** Died | 1,108 (27%) | 1,074 (30%) | 842 (27%) | 1,905 (26%) | 847 (28%) | 787 (33%) | 604 (29%) | 1,408 (27%) | 164 (60%) | 156 (66%) | 104 (64%) | 273 (61%) |
| **Number of Events** |  |  |  |  |  |  |  |  |  |  |  |  |
| 1 | 2,899 (71%) | 2,493 (70%) | 2,132 (68%) | 5,173 (71%) | 2,413 (80%) | 1,899 (79%) | 1,616 (77%) | 4,092 (79%) | 251 (92%) | 226 (95%) | 153 (94%) | 417 (93%) |
| 2 | 744 (18%) | 661 (19%) | 614 (20%) | 1,337 (18%) | 476 (16%) | 386 (16%) | 364 (17%) | 831 (16%) | 19 (6.9%) | 8 (3.4%) | 7 (4.3%) | 26 (5.8%) |
| ≥3 | 417 (10%) | 396 (11%) | 380 (12%) | 730 (10%) | 139 (4.6%) | 114 (4.8%) | 109 (5.2%) | 241 (4.7%) | <5 | <5 | <5 | 7 (1.6%) |
| **History of outcome event***, anytime | 1,597 (39%) | 1,440 (41%) | 1,282 (41%) | 2,675 (37%) | 1,085 (36%) | 738 (31%) | 639 (31%) | 1,580 (31%) | 15 (5.5%) | 11 (4.6%) | 11 (6.8%) | 22 (4.9%) |
| **Socioeconomic Status** |  |  |  |  |  |  |  |  |  |  |  |  |
| 1 (most deprived) | 934 (23%) | 870 (25%) | 758 (24%) | 1,749 (24%) | 724 (24%) | 561 (23%) | 513 (25%) | 1,262 (24%) | 57 (21%) | 48 (20%) | 34 (21%) | 98 (22%) |
| 2 | 1,008 (25%) | 871 (25%) | 737 (24%) | 1,818 (25%) | 731 (24%) | 611 (25%) | 480 (23%) | 1,260 (24%) | 69 (25%) | 79 (33%) | 42 (26%) | 118 (26%) |
| 3 | 890 (22%) | 751 (21%) | 604 (19%) | 1,527 (21%) | 680 (22%) | 498 (21%) | 428 (20%) | 1,102 (21%) | 73 (27%) | 55 (23%) | 37 (23%) | 110 (24%) |
| 4 | 728 (18%) | 622 (18%) | 588 (19%) | 1,275 (18%) | 532 (18%) | 434 (18%) | 384 (18%) | 924 (18%) | 40 (15%) | 26 (11%) | 29 (18%) | 65 (14%) |
| 5 (least deprived) | 494 (12%) | 433 (12%) | 435 (14%) | 864 (12%) | 360 (12%) | 293 (12%) | 281 (13%) | 613 (12%) | 35 (13%) | 30 (13%) | 20 (12%) | 59 (13%) |
| **Body Mass Index** |  |  |  |  |  |  |  |  |  |  |  |  |
| Underweight | 170 (4.2%) | 172 (4.8%) | 139 (4.4%) | 299 (4.1%) | 185 (6.1%) | 181 (7.5%) | 143 (6.8%) | 306 (5.9%) | 8 (2.9%) | 5 (2.1%) | 6 (3.7%) | 15 (3.3%) |
| Normal | 1,299 (32%) | 1,255 (35%) | 1,072 (34%) | 2,399 (33%) | 969 (32%) | 849 (35%) | 733 (35%) | 1,741 (34%) | 62 (23%) | 72 (30%) | 49 (30%) | 126 (28%) |
| Overweight | 945 (23%) | 944 (27%) | 873 (28%) | 1,850 (26%) | 664 (22%) | 606 (25%) | 533 (26%) | 1,227 (24%) | 81 (30%) | 73 (31%) | 56 (35%) | 121 (27%) |
| Obese | 434 (11%) | 450 (13%) | 441 (14%) | 867 (12%) | 273 (9.0%) | 286 (12%) | 281 (13%) | 541 (10%) | 22 (8.0%) | 36 (15%) | 15 (9.3%) | 52 (12%) |
| **Drinking status**, 5yr |  |  |  |  |  |  |  |  |  |  |  |  |
| Drinker | 228 (5.6%) | 211 (5.9%) | 176 (5.6%) | 413 (5.7%) | 215 (7.1%) | 179 (7.5%) | 160 (7.7%) | 375 (7.3%) | 25 (18%) | 16 (11%) | 16 (17%) | 36 (15%) |
| Ex-drinker | 722 (18%) | 821 (23%) | 694 (22%) | 1,423 (20%) | 495 (16%) | 496 (21%) | 403 (19%) | 918 (18%) | 51 (37%) | 60 (43%) | 37 (40%) | 96 (41%) |
| Non-drinker | 1,115 (27%) | 987 (28%) | 888 (28%) | 2,012 (28%) | 792 (26%) | 673 (28%) | 565 (27%) | 1,365 (26%) | 61 (45%) | 64 (46%) | 39 (42%) | 105 (44%) |
| **Smoking status**, 5yr |  |  |  |  |  |  |  |  |  |  |  |  |
| Smoker | 407 (10%) | 368 (10%) | 291 (9.3%) | 737 (10%) | 392 (13%) | 290 (12%) | 260 (12%) | 661 (13%) | 37 (15%) | 28 (12%) | 25 (16%) | 60 (15%) |
| Ex-smoker | 1,266 (31%) | 1,308 (37%) | 1,131 (36%) | 2,415 (33%) | 861 (28%) | 822 (34%) | 711 (34%) | 1,616 (31%) | 96 (40%) | 96 (42%) | 59 (39%) | 161 (40%) |
| Non-smoker | 1,993 (49%) | 1,668 (48%) | 1,514 (48%) | 3,507 (48%) | 1,449 (48%) | 1,164 (49%) | 1,006 (48%) | 2,460 (48%) | 106 (44%) | 106 (46%) | 68 (45%) | 184 (45%) |
| **Systolic BP,** 1yr, mmHg | 135 [124, 144] | 134 [122, 142] | 134 [122, 142] | 135 [124, 143] | 135 [124, 144] | 134 [123, 142] | 134 [122, 142] | 135 [124, 144] | 135  [125, 146] | 134  [123, 142] | 138  [126, 146] | 136 [126, 144] |
| **Diastolic BP**, 1yr, mmHg | 77 [70, 81] | 76 [70, 80] | 76 [70, 80] | 76 [70, 80] | 78 [70, 82] | 76 [70, 80] | 76 [70, 80] | 78 [70, 81] | 78  [70, 80] | 75  [70, 80] | 76  [70, 80] | 76 [70, 80] |
| **No. of medications**, 1yr |  |  |  |  |  |  |  |  |  |  |  |  |
| <10 | 2818 (70%) | 2022 (57%) |  | 4114 (57%) | 2199 (73%) | 1371 (57%) |  | 3075 (60%) | 193 (70%) | 150 (63%) |  | 298 (66%) |
| 10-15 | 848 (21%) | 1,037 (29%) | 2,326 (74%) | 2,326 (32%) | 590 (19%) | 736 (31%) | 1,616 (77%) | 1,616 (31%) | 52 (19%) | 55 (23%) | 113 (70%) | 113 (25%) |
| >15 | 394 (9.7%) | 491 (14%) | 800 (26%) | 800 (11%) | 239 (7.9%) | 292 (12%) | 473 (23%) | 473 (9.2%) | 29 (11%) | 33 (14%) | 49 (30%) | 49 (11%) |
| **Electronic frailty index (eFI)**, 1yr | 2 [1, 3] | 3 [3, 4] | 2 [2, 3] | 2 [1, 3] | 2 [1, 3] | 3 [3, 4] | 2 [2, 3] | 2 [1, 3] | 2 [1, 3] | 3 [3, 4] | 3 [2, 4] | 3 [1, 3] |
| **General practitioner visits**, 1yr | 10 [6, 17] | 13 [9, 20] | 15 [10, 22] | 11 [7, 18] | 10 [5, 16] | 13 [8, 20] | 15 [10, 22] | 11 [6, 17] | 11 [6, 19] | 13 [8, 20] | 6 [11, 22] | 11 [7, 18] |
| **History of comorbidities,** anytime |  |  |  |  |  |  |  |  |  |  |  |  |
| Diabetes | 270 (6.7%) | 577 (16%) | 329 (11%) | 715 (9.9%) | 189 (6.2%) | 370 (15%) | 209 (10%) | 451 (8.7%) | 31 (11%) | 59 (25%) | 27 (17%) | 76 (17%) |
| Vascular disease** | 622 (15%) | 655 (18%) | 544 (17%) | 1,087 (15%) | 386 (13%) | 388 (16%) | 322 (15%) | 655 (13%) | 44 (16%) | 48 (20%) | 34 (21%) | 72 (16%) |
| Chronic renal failure | 454 (11%) | 617 (17%) | 480 (15%) | 943 (13%) | 297 (9.8%) | 396 (17%) | 283 (14%) | 607 (12%) | 52 (19%) | 80 (34%) | 46 (28%) | 110 (24%) |
| Osteoporosis | 546 (13%) | 621 (17%) | 624 (20%) | 1,086 (15%) | 509 (17%) | 526 (22%) | 517 (25%) | 969 (19%) | 27 (9.9%) | 29 (12%) | 17 (10%) | 45 (10%) |

Statistics are presented in N(%) and median [interquartile range].

*falls, fractures and AKI respectively

**Vascular disease include angina pectoris, artery occlusion, atherosclerosis, heart failure, ischemic heart disease, myocardial infarction, peripheral vascular disease, stroke and transient ischemic attack.

BP = blood pressure, ACE-I = angiotensin converting enzyme inhibitor, AKI = acute kidney injury, ARB = angiotensin-ii blocker, BB = beta blocker, CCB = calcium channel blocker,

Missingness of covariates for patients Index of multiple deprivation: <0.1%, BMI 19-37%, Drinking status: 41-50%, Smoking status: 3-13%, systolic BP: 17-39%, diastolic BP: 17-38%

**Table S2: Sensitivity analyses for all antihypertensives and falls**

| **Sensitivity Analyses** | | **Hospitalisation** | | | | **Frailty** | | | | **Polypharmacy** | | | | **Complex Health Needs** | | | |
| --- | --- | --- | --- | --- | --- | --- | --- | --- | --- | --- | --- | --- | --- | --- | --- | --- | --- |
|  | Period | **N** | **FU** | **IRR** | **95%CI** | **N** | **FU** | **IRR** | **95%CI** | **N** | **FU** | **IRR** | **95%CI** | **N** | **FU** | **IRR** | **95%CI** |
| **SCCS assumptions** | | | | | | | | | | | | | | | | | |
| First event only | Unexposed | 3,354 | 15,618 |  |  | 2,878 | 13,259 |  |  | 2,533 | 12,574 |  |  | 5,895 | 28,696 |  |  |
|  | Exposed: 0-30 days | 58 | 205 | 1.17 | 0.89, 1.54 | 58 | 201 | 1.31 | 0.995, 1.71 | 62 | 191 | 1.57 | 1.20, 2.04 | 123 | 399 | 1.41 | 1.17, 1.70 |
|  | Exposed: 31+ days | 648 | 3,865 | 0.84 | 0.74, 0.96 | 614 | 3,480 | 0.99 | 0.86, 1.13 | 531 | 3,320 | 0.94 | 0.81, 1.08 | 1,222 | 7,336 | 0.91 | 0.82, 0.997 |
| Alive during follow-up | Unexposed | 3,441 | 12,383 |  |  | 2,895 | 10,243 |  |  | 2,767 | 10,140 |  |  | 6,114 | 23,083 |  |  |
|  | Exposed: 0-30 days | 55 | 154 | 1.13 | 0.86, 1.50 | 54 | 146 | 1.25 | 0.95, 1.65 | 64 | 143 | 1.53 | 1.18, 1.97 | 118 | 303 | 1.34 | 1.11, 1.62 |
|  | Exposed: 31+ days | 845 | 3,161 | 0.88 | 0.78, 0.999 | 786 | 2,743 | 1.02 | 0.90, 1.16 | 742 | 2,628 | 1.03 | 0.90, 1.17 | 1,575 | 5,930 | 0.96 | 0.88, 1.05 |
| Pre-risk window  (30 days)* | Unexposed | 4,670 | 15,428 |  |  | 4,106 | 13,073 |  |  | 3,692 | 12,396 |  |  | 8,178 | 28,325 |  |  |
|  | Pre-risk window | 106 | 197 | 1.64 | 1.34, 2.01 | 97 | 194 | 1.54 | 1.25, 1.91 | 75 | 185 | 1.30 | 1.02, 1.64 | 184 | 386 | 1.55 | 1.33, 1.81 |
|  | Exposed: 0-30 days | 95 | 205 | 1.41 | 1.14, 1.74 | 92 | 201 | 1.41 | 1.14, 1.75 | 94 | 191 | 1.56 | 1.26, 1.93 | 181 | 399 | 1.47 | 1.26, 1.71 |
|  | Exposed: 31+ days | 1,124 | 3,857 | 0.97 | 0.87, 1.08 | 1,073 | 3,472 | 1.05 | 0.94, 1.18 | 1,001 | 3,313 | 1.06 | 0.95, 1.18 | 2,097 | 7,321 | 1.02 | 0.94, 1.10 |
| **Other** | | | | | | | | | | | | | | | | | |
| No history of falls | Unexposed | 2,617 | 10,096 |  |  | 2,272 | 8,403 |  |  | 1,927 | 7,926 |  |  | 4,777 | 19,134 |  |  |
|  | Exposed: 0-30 days | 59 | 128 | 1.53 | 1.17, 2.01 | 50 | 124 | 1.43 | 1.07, 1.91 | 58 | 117 | 1.86 | 1.42, 2.45 | 115 | 259 | 1.61 | 1.32, 1.95 |
|  | Exposed: 31+ days | 641 | 2,528 | 0.89 | 0.77, 1.02 | 609 | 2,263 | 1.01 | 0.88, 1.17 | 573 | 2,128 | 1.12 | 0.96, 1.29 | 1,277 | 4,959 | 0.995 | 0.90, 1.1 |
| AH gap length 180 days | Unexposed | 4,764 | 15,595 |  |  | 4,192 | 13,232 |  |  | 3,759 | 12,548 |  |  | 8,345 | 28,647 |  |  |
|  | Exposed: 0-30 days | 88 | 182 | 1.40 | 1.12, 1.74 | 81 | 176 | 1.37 | 1.08, 1.70 | 83 | 168 | 1.52 | 1.21, 1.90 | 162 | 353 | 1.42 | 1.21, 1.67 |
|  | Exposed: 31+ days | 1,143 | 3,911 | 0.93 | 0.84, 1.03 | 1,095 | 3,532 | 1.01 | 0.91, 1.13 | 1,020 | 3,368 | 1.04 | 0.93, 1.16 | 2,133 | 7,432 | 0.97 | 0.90, 1.05 |

N = Number of falls, FU = Follow-up in patient years, IRR = Incidence rate ratio, 95%CI = 95% confidence interval, AH = Antihypertensives

*For people with multiple exposed periods, introducing the pre-risk window can lead to an overlap between the end of the previous exposed period and the pre-risk window, with the events being included in the pre-risk window only.

**Table S3: SCCS main analyses stratified for AH drug class and falls**

|  | | **HOSPITALISATION** | | | | **FRAILTY** | | | | **POLYPHARMACY** | | | | **Complex Health Needs** | | | |
| --- | --- | --- | --- | --- | --- | --- | --- | --- | --- | --- | --- | --- | --- | --- | --- | --- | --- |
|  |  | **N** | **FU** | **IRR** | **95%CI** | **N** | **FU** | **IRR** | **95%CI** | **N** | **FU** | **IRR** | **95%CI** | **N** | **FU** | **IRR** | **95%CI** |
| ACE-I | Unexposed | 3,808 | 12,181 |  |  | 3,255 | 10,082 |  |  | 2,799 | 9,165 |  |  | 6,464 | 21,774 |  |  |
|  | Exposed: 0-30 days | 20 | 52 | 1.24 | 0.79, 1.95 | 24 | 55 | 1.57 | 1.03, 2.37 | 15 | 45 | 1.17 | 0.7, 1.97 | 37 | 105 | 1.25 | 0.9, 1.75 |
|  | Exposed: 31+ days | 273 | 1,013 | 0.88 | 0.73, 1.06 | 318 | 1,024 | 1.2 | 0.996, 1.43 | 239 | 876 | 1.01 | 0.83, 1.24 | 551 | 2,035 | 1.01 | 0.88, 1.16 |
| ARB | Unexposed | 3,395 | 10,642 |  |  | 2,886 | 8,556 |  |  | 2,461 | 7,815 |  |  | 5,741 | 18,782 |  |  |
|  | Exposed: 0-30 days | <5 | NA | NA | NA | 0 | 13 | 0 | 0, Inf | 0 | 14 | 0 | 0, Inf | <5 | NA | NA | NA |
|  | Exposed: 31+ days | 75 | 328 | 0.83 | 0.57, 1.19 | 72 | 306 | 0.77 | 0.53, 1.10 | 69 | 319 | 0.67 | 0.47, 0.95 | 154 | 659 | 0.85 | 0.66, 1.09 |
| BB | Unexposed | 3,793 | 12,193 |  |  | 3,211 | 9,782 |  |  | 2,820 | 9,150 |  |  | 6,439 | 21,548 |  |  |
|  | Exposed: 0-30 days | 19 | 53 | 1.2 | 0.75, 1.9 | 25 | 46 | 1.75 | 1.17, 2.63 | 19 | 44 | 1.40 | 0.88, 2.23 | 39 | 99 | 1.31 | 0.95, 1.82 |
|  | Exposed: 31+ days | 318 | 1,067 | 1.09 | 0.91, 1.31 | 296 | 905 | 1.15 | 0.95, 1.4 | 253 | 867 | 1.03 | 0.84, 1.26 | 568 | 1,971 | 1.02 | 0.89, 1.17 |
| CCB | Unexposed | 3,902 | 12,582 |  |  | 3,359 | 10,334 |  |  | 2,916 | 9,581 |  |  | 6,691 | 22,586 |  |  |
|  | Exposed: 0-30 days | 29 | 67 | 1.47 | 1.01, 2.14 | 22 | 60 | 1.18 | 0.77, 1.81 | 22 | 57 | 1.34 | 0.87, 2.05 | 50 | 127 | 1.38 | 1.03, 1.83 |
|  | Exposed: 31+ days | 269 | 1,181 | 0.74 | 0.61, 0.88 | 226 | 963 | 0.74 | 0.61, 0.89 | 234 | 920 | 0.85 | 0.7, 1.03 | 516 | 2,194 | 0.80 | 0.70, 0.91 |
| Diuretics | Unexposed | 4,425 | 14,179 |  |  | 3,915 | 12,196 |  |  | 3,466 | 11,402 |  |  | 7,731 | 26,041 |  |  |
|  | Exposed: 0-30 days | 61 | 128 | 1.32 | 1.02, 1.72 | 72 | 135 | 1.58 | 1.24, 2.01 | 72 | 128 | 1.72 | 1.35, 2.2 | 128 | 257 | 1.50 | 1.25, 1.80 |
|  | Exposed: 31+ days | 513 | 1,511 | 1.01 | 0.89, 1.16 | 516 | 1,448 | 1.13 | 0.98, 1.29 | 487 | 1,403 | 1.17 | 1.02, 1.35 | 954 | 2,931 | 1.06 | 0.96, 1.17 |

Analyses were conducted separately for each antihypertensive drug class, no co-medication (incl. other antihypertensive drug classes) was included.

N = Number of falls, FU = Follow-up in patient years, IRR = Incidence rate ratio, 95%CI = 95% confidence interval, ACE-I = angiotensin-converting-enzyme inhibitors, ARB = angiotensin-II receptor blockers, BB = beta blockers, CCB = calcium-channel blockers

Cell counts <5 are clouded as required by database policies and no associated IRR is presented.

**Table S4: Sensitivity analyses for all antihypertensives and fractures**

| **Sensitivity Analyses** | | **Hospitalisation** | | | | **Frailty** | | | | **Polypharmacy** | | | | **Complex Health Needs** | | | |
| --- | --- | --- | --- | --- | --- | --- | --- | --- | --- | --- | --- | --- | --- | --- | --- | --- | --- |
|  | Period | **N** | **FU** | **IRR** | **95%CI** | **N** | **FU** | **IRR** | **95%CI** | **N** | **FU** | **IRR** | **95%CI** | **N** | **FU** | **IRR** | **95%CI** |
| **SCCS assumptions** | | | | | | | | | | | | | | | | | |
| First event only | Unexposed | 2,492 | 11,877 |  |  | 1,925 | 9,131 |  |  | 1,637 | 8,475 |  |  | 4,181 | 21,010 |  |  |
|  | Exposed: 0-30 days | 41 | 147 | 1.20 | 0.87, 1.65 | 46 | 136 | 1.53 | 1.12, 2.08 | 38 | 130 | 1.49 | 1.07, 2.09 | 76 | 273 | 1.32 | 1.04, 1.68 |
|  | Exposed: 31+ days | 495 | 2,845 | 0.91 | 0.78, 1.06 | 428 | 2,304 | 1.02 | 0.86, 1.20 | 414 | 2,283 | 1.05 | 0.88, 1.24 | 907 | 5,069 | 1.01 | 0.90, 1.14 |
| Alive during follow-up | Unexposed | 2,241 | 9,438 |  |  | 1,632 | 6,950 |  |  | 1,514 | 6,724 |  |  | 3,829 | 16,855 |  |  |
|  | Exposed: 0-30 days | 37 | 108 | 1.32 | 0.94, 1.85 | 32 | 96 | 1.41 | 0.98, 2.03 | 32 | 97 | 1.46 | 1.02, 2.10 | 62 | 204 | 1.27 | 0.98, 1.65 |
|  | Exposed: 31+ days | 508 | 2,254 | 0.96 | 0.82, 1.12 | 432 | 1,757 | 1.13 | 0.95, 1.34 | 423 | 1,755 | 1.10 | 0.92, 1.31 | 931 | 3,996 | 1.06 | 0.94, 1.19 |
| Pre-risk window (30 days)* | Unexposed | 3,040 | 11,740 |  |  | 2,353 | 9,005 |  |  | 2,053 | 8,354 |  |  | 5,122 | 20,756 |  |  |
|  | Pre-risk window | 54 | 142 | 1.33 | 1.00, 1.76 | 47 | 131 | 1.35 | 0.995, 1.82 | 38 | 125 | 1.20 | 0.86, 1.68 | 100 | 264 | 1.45 | 1.18, 1.78 |
|  | Exposed: 0-30 days | 53 | 147 | 1.24 | 0.94, 1.65 | 52 | 136 | 1.41 | 1.06, 1.89 | 42 | 130 | 1.28 | 0.93, 1.75 | 91 | 273 | 1.27 | 1.02, 1.57 |
|  | Exposed: 31+ days | 690 | 2,840 | 0.96 | 0.84, 1.10 | 610 | 2,299 | 1.12 | 0.97, 1.30 | 581 | 2,278 | 1.07 | 0.92, 1.25 | 1,255 | 5,059 | 1.07 | 0.96, 1.18 |
| **Fracture type** | | | | | | | | | | | | | | | | | |
| Hip fracture | Unexposed | 850 | 3,287 |  |  | 746 | 2,994 |  |  | 593 | 2,546 |  |  | 1,469 | 6,147 |  |  |
|  | Exposed: 0-30 days | 21 | 45 | 1.59 | 0.997, 2.54 | 13 | 47 | 0.99 | 0.55, 1.77 | 8 | 42 | 0.80 | 0.39, 1.65 | 26 | 86 | 1.14 | 0.76, 1.71 |
|  | Exposed: 31+ days | 164 | 703 | 0.94 | 0.72, 1.23 | 180 | 669 | 1.19 | 0.92, 1.56 | 149 | 646 | 1.12 | 0.85, 1.49 | 326 | 1,336 | 1.09 | 0.9, 1.32 |
| Vertebral fracture** | Unexposed | 201 | 967 |  |  | 179 | 825 |  |  | 189 | 965 |  |  | 394 | 1,969 |  |  |
|  | Exposed: 0-30 days | <5 | NA | NA | NA | <5 | NA | NA | NA | 7 | 15 | 2.18 | 0.99, 4.82 | 8 | 28 | 1.34 | 0.65, 2.76 |
|  | Exposed: 31+ days | 49 | 214 | 1.33 | 0.82, 2.13 | 59 | 266 | 1.51 | 0.98, 2.33 | 55 | 255 | 1.31 | 0.84, 2.05 | 115 | 530 | 1.42 | 1.05, 1.94 |
| Non-hip-non-vertebral fracture | Unexposed | 2,339 | 9,703 |  |  | 1,734 | 7,209 |  |  | 1,531 | 6,810 |  |  | 3,896 | 16,981 |  |  |
|  | Exposed: 0-30 days | 38 | 122 | 1.15 | 0.82, 1.60 | 39 | 106 | 1.46 | 1.04, 2.04 | 30 | 102 | 1.29 | 0.89, 1.87 | 66 | 218 | 1.22 | 0.95, 1.57 |
|  | Exposed: 31+ days | 535 | 2,460 | 0.87 | 0.75, 1.02 | 434 | 1,858 | 1.01 | 0.85, 1.2 | 446 | 1,871 | 1.10 | 0.93, 1.31 | 940 | 4,218 | 0.99 | 0.88, 1.11 |
| Serious fracture (hospital) | Unexposed | 1,663 | 6,706 |  |  | 1,327 | 5,360 |  |  | 1,159 | 5,008 |  |  | 2,825 | 12,020 |  |  |
|  | Exposed: 0-30 days | 34 | 88 | 1.40 | 0.98, 2.01 | 24 | 83 | 1.08 | 0.70, 1.65 | 20 | 80 | 1.05 | 0.66, 1.65 | 52 | 165 | 1.24 | 0.93, 1.65 |
|  | Exposed: 31+ days | 381 | 1,669 | 0.93 | 0.77, 1.12 | 353 | 1,372 | 1.17 | 0.96, 1.42 | 314 | 1,424 | 0.94 | 0.76, 1.15 | 689 | 2,985 | 1.02 | 0.89, 1.17 |
| **Other** | |  |  |  |  |  |  |  |  |  |  |  |  |  |  |  |  |
| No history of fractures | Unexposed | 1,897 | 7,822 |  |  | 1,640 | 6,540 |  |  | 1,400 | 6,055 |  |  | 3,534 | 14,974 |  |  |
|  | Exposed: 0-30 days | 30 | 97 | 1.08 | 0.74, 1.58 | 30 | 93 | 1.24 | 0.85, 1.80 | 27 | 91 | 1.29 | 0.87, 1.91 | 55 | 192 | 1.11 | 0.85, 1.47 |
|  | Exposed: 31+ days | 464 | 2,011 | 0.90 | 0.76, 1.07 | 415 | 1,624 | 1.08 | 0.91, 1.29 | 414 | 1,669 | 1.16 | 0.97, 1.39 | 890 | 3,710 | 1.04 | 0.92, 1.17 |
| Fracture gap length 90days | Unexposed | 2,887 | 11,877 |  |  | 2,242 | 9,131 |  |  | 1,946 | 8,475 |  |  | 4,886 | 21,010 |  |  |
|  | Exposed: 0-30 days | 51 | 147 | 1.26 | 0.94, 1.68 | 49 | 136 | 1.36 | 1.01, 1.83 | 40 | 130 | 1.27 | 0.92, 1.76 | 88 | 273 | 1.27 | 1.02, 1.58 |
|  | Exposed: 31+ days | 636 | 2,845 | 0.94 | 0.82, 1.08 | 569 | 2,304 | 1.08 | 0.93, 1.25 | 541 | 2,283 | 1.04 | 0.89, 1.22 | 1,170 | 5,069 | 1.03 | 0.93, 1.14 |
| AH gap length 180days | Unexposed | 3,085 | 11,860 |  |  | 2,395 | 9,111 |  |  | 2,085 | 8,459 |  |  | 5,212 | 20,977 |  |  |
|  | Exposed: 0-30 days | 48 | 131 | 1.24 | 0.92, 1.66 | 44 | 118 | 1.33 | 0.97, 1.81 | 37 | 115 | 1.27 | 0.91, 1.78 | 81 | 242 | 1.23 | 0.98, 1.54 |
|  | Exposed: 31+ days | 704 | 2,879 | 0.95 | 0.83, 1.09 | 623 | 2,341 | 1.09 | 0.94, 1.26 | 592 | 2,314 | 1.06 | 0.91, 1.23 | 1,275 | 5,134 | 1.03 | 0.93, 1.14 |

N = Number of fractures, FU = Follow-up in patient years, IRR = Incidence rate ratio, 95%CI = 95% confidence interval, AH = Antihypertensives

*For people with multiple exposed periods, introducing the pre-risk window can lead to an overlap between the end of the previous exposed period and the pre-risk window, with the events being included in the pre-risk window only. **As for the small cell counts, this analysis was NOT adjusted for age. Cell counts <5 are clouded as required by database policies and no associated IRR is presented.

**Table S5: SCCS main analyses stratified for AH drug class and fractures**

|  | | **HOSPITALISATION** | | | | **FRAILTY** | | | | **POLYPHARMACY** | | | | **Complex Health Needs** | | | |
| --- | --- | --- | --- | --- | --- | --- | --- | --- | --- | --- | --- | --- | --- | --- | --- | --- | --- |
|  |  | **N** | **FU** | **IRR** | **95%CI** | **N** | **FU** | **IRR** | **95%CI** | **N** | **FU** | **IRR** | **95%CI** | **N** | **FU** | **IRR** | **95%CI** |
| ACE-I | Unexposed | 2,453 | 9,237 |  |  | 1,896 | 6,889 |  |  | 1,570 | 6,052 |  |  | 4,084 | 15,963 |  |  |
|  | Exposed: 0-30 days | 12 | 38 | 1.29 | 0.72, 2.32 | 14 | 34 | 1.67 | 0.97, 2.89 | 13 | 29 | 2.05 | 1.16, 3.606 | 22 | 69 | 1.39 | 0.9, 2.13 |
|  | Exposed: 31+ days | 183 | 791 | 1.04 | 0.82, 1.32 | 149 | 632 | 1.08 | 0.84, 1.4 | 134 | 558 | 1.14 | 0.859, 1.517 | 330 | 1,405 | 1.12 | 0.94, 1.34 |
| ARB | Unexposed | 2,222 | 8,139 |  |  | 1,695 | 5,907 |  |  | 1,391 | 5,149 |  |  | 3,675 | 13,914 |  |  |
|  | Exposed: 0-30 days | 5 | 10 | 2.31 | 0.92, 5.78 | <5 | NA | NA | NA | <5 | NA | NA | NA | 6 | 19 | 1.47 | 0.65, 3.35 |
|  | Exposed: 31+ days | 45 | 215 | 0.94 | 0.6, 1.5 | 43 | 207 | 0.8 | 0.49, 1.3 | 41 | 194 | 0.93 | 0.568, 1.517 | 98 | 433 | 1.03 | 0.75, 1.43 |
| BB | Unexposed | 2,467 | 9,272 |  |  | 1,882 | 6,767 |  |  | 1,604 | 6,145 |  |  | 4,108 | 15,928 |  |  |
|  | Exposed: 0-30 days | 15 | 39 | 1.38 | 0.81, 2.37 | 11 | 31 | 1.16 | 0.62, 2.18 | 5 | 31 | 0.63 | 0.258, 1.528 | 17 | 69 | 0.89 | 0.54, 1.45 |
|  | Exposed: 31+ days | 201 | 788 | 1.09 | 0.87, 1.38 | 160 | 574 | 1.08 | 0.83, 1.39 | 150 | 610 | 0.98 | 0.749, 1.272 | 340 | 1,349 | 1.04 | 0.87, 1.24 |
| CCB | Unexposed | 2,527 | 9,604 |  |  | 1,915 | 7,129 |  |  | 1,617 | 6,436 |  |  | 4,206 | 16,689 |  |  |
|  | Exposed: 0-30 days | 8 | 48 | 0.7 | 0.35, 1.41 | 14 | 41 | 1.61 | 0.94, 2.77 | 11 | 40 | 1.38 | 0.75, 2.529 | 23 | 88 | 1.18 | 0.78, 1.80 |
|  | Exposed: 31+ days | 188 | 891 | 0.96 | 0.76, 1.19 | 160 | 636 | 1.25 | 0.97, 1.59 | 172 | 658 | 1.39 | 1.091, 1.772 | 348 | 1,564 | 1.05 | 0.89, 1.24 |
| Diuretics | Unexposed | 2,872 | 10,747 |  |  | 2,270 | 8,391 |  |  | 1,987 | 7,736 |  |  | 4,868 | 19,065 |  |  |
|  | Exposed: 0-30 days | 39 | 90 | 1.35 | 0.97, 1.88 | 38 | 91 | 1.43 | 1.02, 1.99 | 35 | 88 | 1.37 | 0.967, 1.936 | 73 | 175 | 1.44 | 1.13, 1.83 |
|  | Exposed: 31+ days | 285 | 1,091 | 0.85 | 0.71, 1.02 | 286 | 986 | 1.04 | 0.86, 1.24 | 267 | 998 | 0.91 | 0.754, 1.097 | 540 | 2,006 | 0.98 | 0.86, 1.11 |

Analyses were conducted separately for each antihypertensive drug class, no co-medication (incl. other antihypertensive drug classes) was included.

N = Number of falls, FU = Follow-up in patient years, IRR = Incidence rate ratio, 95%CI = 95% confidence interval, ACE-I = angiotensin-converting-enzyme inhibitors, ARB = angiotensin-II receptor blockers, BB = beta blockers, CCB = calcium-channel blockers.

Cell counts <5 are clouded as required by database policies and no associated IRR is presented.

**Table S6: Sensitivity analyses for all antihypertensives and acute kidney injury**

| **Sensitivity Analyses** | | **Hospitalisation** | | | | **Frailty** | | | | **Polypharmacy** | | | | **Complex Health Needs** | | | |
| --- | --- | --- | --- | --- | --- | --- | --- | --- | --- | --- | --- | --- | --- | --- | --- | --- | --- |
|  | Period | **N** | **FU** | **IRR** | **95%CI** | **N** | **FU** | **IRR** | **95%CI** | **N** | **FU** | **IRR** | **95%CI** | **N** | **FU** | **IRR** | **95%CI** |
| **SCCS assumptions** | |  |  |  |  |  |  |  |  |  |  |  |  |  |  |  |  |
| First event only | Unexposed | 176 | 806 |  |  | 145 | 696 |  |  | 95 | 462 |  |  | 272 | 1,358 |  |  |
|  | Exposed | 98 | 300 | 2.18 | 1.38, 3.44 | 93 | 327 | 2.70 | 1.57, 4.62 | 67 | 217 | 3.06 | 1.67, 5.61 | 178 | 609 | 2.58 | 1.81, 3.69 |
| Alive during follow-up | Unexposed | 84 | 425 |  |  | 46 | 306 |  |  | 34 | 210 |  |  | 115 | 676 |  |  |
|  | Exposed | 37 | 159 | 1.02 | 0.54, 1.91 | 40 | 172 | 1.84 | 0.79, 4.30 | 27 | 108 | 2.02 | 0.86, 4.73 | 76 | 338 | 1.39 | 0.86, 2.25 |
| Pre-risk window (30 days)* | Unexposed | 194 | 808 |  |  | 148 | 701 |  |  | 100 | 455 |  |  | 291 | 1,371 |  |  |
|  | Pre-risk window | <5 | NA | NA | NA | <5 | NA | NA | NA | 5 | 11 | 3.24 | 1.19, 8.80 | 9 | 29 | 2.01 | 0.99, 4.08 |
|  | Exposed | 105 | 299 | 1.97 | 1.26, 3.08 | 102 | 322 | 2.87 | 1.69, 4.89 | 68 | 216 | 3.48 | 1.90, 6.37 | 191 | 602 | 2.55 | 1.80, 3.60 |
| **Other** | |  |  |  |  |  |  |  |  |  |  |  |  |  |  |  |  |
| No history of AKI | Unexposed | 182 | 794 |  |  | 142 | 688 |  |  | 96 | 441 |  |  | 281 | 1,349 |  |  |
|  | Exposed | 100 | 289 | 1.80 | 1.15, 2.81 | 98 | 317 | 2.49 | 1.46, 4.26 | 62 | 203 | 2.62 | 1.41, 4.87 | 183 | 581 | 2.24 | 1.58, 3.17 |
| AH gap length 180 days | Unexposed | 196 | 816 |  |  | 151 | 708 |  |  | 105 | 465 |  |  | 298 | 1,384 |  |  |
|  | Exposed | 106 | 302 | 1.93 | 1.25, 2.98 | 103 | 324 | 2.65 | 1.57, 4.45 | 68 | 218 | 2.85 | 1.59, 5.10 | 193 | 607 | 2.30 | 1.64, 3.22 |

N = Number of acute kidney injury, FU = Follow-up in patient years, IRR = Incidence rate ratio, 95%CI = 95% confidence interval. AKI = severe acute kidney injury, AH = Antihypertensives

Cell counts <5 are clouded as required by database policies and no associated IRR is presented.

*For people with multiple exposed periods, introducing the pre-risk window can lead to an overlap between the end of the previous exposed period and the pre-risk window, with the events being included in the pre-risk window only.
